# Exome Sequencing: Mutilating Sensory Neuropathy with Spastic Paraplegia due to a Mutation in FAM134B Gene

**DOI:** 10.1155/2018/9468049

**Published:** 2018-12-12

**Authors:** Salma M. Wakil, Dorota Monies, Samya Hagos, Fahad Al-Ajlan, Josef Finsterer, Aisha Al Qahtani, Khushnooda Ramzan, Rawan Al Humaidy, Mohamed A. Al-Muhaizea, Brian Meyer, Saeed A. Bohlega

**Affiliations:** ^1^Department of Genetics, King Faisal Specialist Hospital and Research Centre, Riyadh, Saudi Arabia; ^2^Department of Neurology, King Faisal Specialist Hospital and Research Centre, Riyadh, Saudi Arabia; ^3^Department of Neurology Krankenanstalt Rudolfstiftung, Vienna, Austria

## Abstract

Hereditary sensory and autonomic neuropathies (HSANs) are a clinically and genetically heterogeneous group of disorders involving various sensory and autonomic dysfunctions. The most common symptoms of HSANs include loss of sensations of pain and temperature that frequently lead to chronic ulcerations in the feet and hands of the patient. In this case study, we present the clinical features and genetic characteristics of two affected individuals from two unrelated Saudi families presenting mutilating sensory loss and spastic paraplegia. We employed homozygosity mapping and exome sequencing which is an efficient strategy to characterize the recessive genes, thus obtaining a rapid molecular diagnosis for genetically heterogeneous disorders like HSAN. Subsequently, a nonsense mutation (c.926 C>G; p.S309⁎) in* FAM134B *was identified. In addition, we confirmed that the mutant FAM134B transcripts were reduced in these patients presumably disrupting the receptors of the degradative endoplasmic reticulum pathways that facilitate the autophagy processes.

## 1. Background

Hereditary sensory and autonomic neuropathies (HSANs) are clinically and genetically heterogeneous group of disorders of the peripheral nervous system with various patterns of inheritance. The term HSAN was originally described in 1979 by Cavanagh et al. who presented five cases of early onset spastic paraplegia with sensory neuropathy [1]. Spastic paraplegia in their study was mild and had been overshadowed by much more striking mutilating lower limb acropathies. Nerve biopsy in HSAN shows axonopathy with loss of myelinated and unmyelinated nerve fibers of all diameters but with normal appearance of brain and spinal cord [2]. HSAN-II is predominantly a sensory neuropathy that shows autosomal recessive inheritance; four types of HSAN-II (2A, 2B, 2C, and 2D) have been described. Patients with HSAN2B (MIM#613115) is characterized with the presence of pronounced autonomic dysfunction with or without distal motor involvement as compared to HSAN2A while in HSAN2C patients are accompanied with distal muscle weakness. HSAN2D is characterized with insensitivity to pain but otherwise normal sensory modalities. HSAN2B manifests in infancy or early childhood with distal numbness and progressive loss of pain, temperature, and touch sensation [3]. Here we describe two unrelated consanguineous Saudi families presenting HSAN2B with paraspasticity caused by a* FAM134B *mutation. Five mutations in* FAM134B *have been reported to cause HSAN2B (Human Gene Mutation Database; http://www.hgmd.cf.ac.uk). Further we confirmed that the mutant FAM134B transcripts were reduced in these patients presumably disrupting the receptors of degradative endoplasmic reticulum (ER) pathways that facilitate the autophagy processes.

## 2. Case Presentation

Two families were recruited in the neurology clinic at King Faisal Specialist Hospital & Research Center (KFSH&RC), Riyadh, Saudi Arabia. All subjects (patients and unaffected family members) were enrolled under an IRB-approved protocol (RAC#2090011).

**Figure 1:**
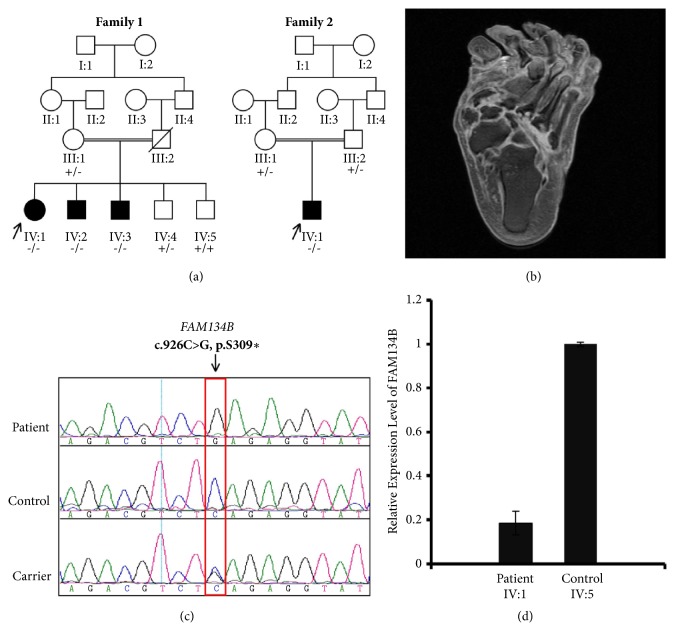
(a) Pedigree of the described Family 1. Parents (III: 1 and III: 2) of the affected individuals (IV: 1, IV: 2 and IV: 3) are first degree relatives. Family 2 Parents (III: 1 and III: 2) of the affected individuals (IV: 1) are first degree relatives. Filled boxes indicate individuals affected by hereditary sensory neuropathy and open boxes indicate phenotypically normal individuals. Homozygous mutant genotype (-/-), heterozygous carrier (**+**/-), and homozygous wild-type genotype (**+**/**+**) were observed. (b) MRI of the foot for the affected individual IV: 1 from Family 1. Note the marked deformity in the toe with metatarsal bone and scar tissue formation. (c) Sanger sequencing analysis of the* FAM134B *gene (NM_ 001034850) identified a homozygous variant (c.926C>G, p.S309*∗*) in the affected individual (upper panel), homozygous wild type in the normal control (middle panel) and heterozygous in the carrier mother (lower panel). (d) Quantitative real time PCR of* FAM134B *mRNA for patient IV: 1 from family 1 and his normal sibling IV: 2 showing significant reduction of the transcript level. The experiment was performed in quadruplicate; error bars represent standard error of the mean while the relative expression of* FAM134B *in controls is set to one and data are normalized to GAPDH mRNA.

**Family 1: **Three affected children (F1:IV:1, IV:2, and IV:3) presented HSAN2B phenotype with slightly variable age of onset, disease course, and severity [Figure 1(a)]. As per the clinical history obtained from the family, the initial clinical manifestations were spastic scissoring gait and frequent falls between the ages of one and three years. Multiple painless ulcers developed at the base of both big toes and soles with recurrent soft tissue and bone infections leading to acropathies and deformities of the feet and lower legs [Figure 1(b)]. During 30 years of follow-up at multiple hospitals, the index case IV:1 was admitted several times for treating mutilating ulcers and deformities. There was mild weakness in feet and legs (MRC4). Hyperreflexia with clonus in lower limbs was also noted. Abdominal reflexes and deep tendon reflexes in the upper limbs were normal. Spinal MRI showed mild thinning of the spinal cord mainly on the thoracic region. School performance was acceptable and two of the patients IV:2 and IV:3 held permanent jobs.

**Family 2: **A 15-year-old boy (F2: IV: 1) presented with a history of frequent falls, unsteadiness, and pain insensitivity from an early age of 4 years [Figure 1(a)]. During the 5-year follow-up, he was hospitalized multiple times due to skin ulcers and osteomyelitis affecting his feet and toes. There was mild spasticity in the lower limbs with minimal pyramidal weakness (MRC4). Tendon reflexes were exaggerated with negative extensor response.

Touch, pinprick, temperature and vibration revealed mild impairment in the distal part of the lower extremities for all the affected's from family 1 while it was normal for family 2. In both families, applying strong pressure to the Achilles tendon or touching the exposed bony areas was not followed by an adequate pain reflex. Neurophysiological findings were normal or mildly abnormal in family 1 in the early stages but follow-up studies revealed sensory axonal polyneuropathy predominantly in the lower limbs while it was normal for family 2. Sympathetic skin response and beat to beat variation were also found to be normal for family 2 but abnormal in family 1 indicating involvement of the autonomic nervous system. Cerebral MRI as well as other hematological and biochemical investigations was normal in both the families (Table 1).

## 3. Genetic Evaluation

Genomic DNA was extracted from peripheral blood samples using standard salt precipitation procedures. Samples were quantitated spectrophotometrically and stored at -20°C. Genome wide single nucleotide polymorphism (SNP) genotyping was performed using Axiom Genome-Wide ASI Array's and Gene Titan MC Instrument (Affymetrix, Santa Clara, Inc. USA) providing a high genetic coverage of 587,352 SNPs across the whole genome. Genomic DNA from all the individuals of family 1 was used for SNP genotyping and to detect regions of homozygosities (ROH), and analysis was performed by AutoSNPa software. ROH are defined as long contiguous stretches of genotypes that are identical, or homozygous, in affected individuals due to the transmission of identical genotypes from parents to their children. A ROH (39-Mb) shared exclusively by affected siblings of family 1 was identified on chromosome 5p15.2-q11.3 flanked by rs835062 and rs12516870. Whole-exome sequencing (WES) was carried out for the index case of family1 (IV: 1) to identify the likely causal variant. For WES, briefly 2 microgram of DNA was used to obtain the Ion proton AmpliSeq library. Amplified exome targets were ligated with Ion P1 and Ion Xpress Barcode adapters. After purification, the libraries were quantified using qPCR with the Ion Library Quantification Kit (Thermo Fisher, Carlsbad, CA, USA). The prepared exome libraries underwent emulsion PCR following the manufacturer's instructions (Thermo Fisher, Carlsbad, CA, USA). Finally the template-positive PI Ion Sphere particles were processed for sequencing on the Ion Proton instrument. Resulting data was analyzed and annotated with reads mapped to UCSC hg19 (http://genome.ucsc.edu/). We identified a total of 40,000 homozygous variants relative to hg19 with 250 being present within the predefined autozygome on chromosome 5. These variants were further filtered on the basis of MAF < 0.001 and only one variant (c.926C>G; p.S309*∗*) in* FAM134B *(NM_001034850) survived the filtration. To validate the WES identified variant, mutational screening was carried out by PCR amplification using standard protocols and primers were designed using primer3 (http://frodo.wi.Mit.edu/primer3/). PCR amplicons were purified and sequenced with ABI PRISM Big Dye Terminator v3.1 Cycle Sequencing Kit on an ABI PRISM 3730 DNA Analyzer (Applied Biosystems, Inc., Foster City, CA, USA). Sequence analysis was performed using Lasergene (DNA Star Inc., Madison, WI, USA) software package. Segregation of the sequence variant with the disease phenotype was done for all the members in the family. Due to similar phenotype, the index case (IV: 1) and parents from family 2 were also Sanger sequenced for WES-identified variant, which fully segregated with disease phenotype in both families [Figures 1(a) and 1(c)]. To investigate the effect of nonsense mutation at the transcript level, we carried out real time PCR quantification of FAM134B mRNA in the patient (IV: 1; family 1) and homozygous wildtype sibling (IV: 5; family 1) as a control. Total RNA was extracted from Paxgene tubes using Blood RNA Kit (PreAnalytix, Qiagen) which was reverse transcribed using iScript cDNA synthesis kit (Biorad). The quantitative PCR was performed on the synthesized cDNA using Platinum SYBR Green qPCR SuperMix-UDG kit (Invitrogen) and Applied Biosystems Real Time PCR 7500 Fast system. Real time quantification of* FAM134B *mRNA in the index patient (IV: 1) of family 1 indicated a 4-fold reduction in* FAM134B *mRNA expression compared to the normal sibling [Figure 1(d)].

## 4. Discussion and Conclusion

HSAN2B is a predominantly sensory neuropathy and has been rarely described with spastic paraplegia. Among previously reported cases, the disease progression was highly variable. Our patients presented with an early disease onset with mild spasticity and weakness of the lower limbs, followed by ulceration and infection of extremities due to pain insensitivity leading to mutilating sensory neuropathy. Neurophysiological findings were normal or mildly abnormal in the early stages but later on sensory axonal polyneuropathy mainly involving the lower extremities developed. Lower leg spasticity and weakness did not progress remarkably and all patients remained ambulatory for many years. These patients clinically resembled cases with mutilating hereditary sensory neuropathy and spastic paraplegia from Turkish and Moroccan ancestry with different variants in* FAM134B *(p.S276Vfs*∗*8) and* Cct5 *(p.H147R) genes, respectively [4–6]. Additionally, two cousins of Turkish ancestry following differing clinical courses of hereditary sensory and autonomic neuropathy were reported with unidentified molecular variant. All the known genes of HSAN2B including* FAM134B *were found be negative, thus postulating that there could be an additional novel gene for the Turkish phenotype [7]. While the pathogenic variant identified in our study has been previously reported [8], in addition our patients also exhibited spastic paraplegia.* FAM134B *encodes newly identified Golgi proteins and belongs to a family of three genes namely* FAM134A*,* FAM134B, *and* FAM134C*. Proteins of FAM134 family are involved in selective autophagy, a degradative pathway which is involved in turnover of specific proteins and organelles, and FAM134 proteins colocalize with cis-Golgi marker giantin. It has been shown that the members of this family protein upon binding to autophagy receptors modulate ER degradation [9]. Any aberration inFAM134B will perturb the dynamic shape of Golgi apparatus which is essential for the processing and distributions of lipids and proteins. Accordingly, the abnormal FAM134B proteins are unable to act as ER-phagy receptors that compromise survival of sensory and autonomic neurons and progressive neurodegeneration. The mutation p.S309*∗* is predicted to result in nonsense-mediated decay with complete absence of the protein or to a truncated and probably nonfunctional gene product. The downregulation of FAM134B protein further supports the proposed loss of its function disrupting the ER mechanism underlying the HSAN type II. However the pathogenetic role of* FAM134B *in sensory neuropathy with spastic paraplegia remains largely unknown and this study expands the phenotypic heterogeneity caused due to variants in* FAM134B*. Homozygosity mapping and exome sequencing were employed to identify the precise molecular genetic diagnosis in our study as an efficient strategy. Overall, this study is confirming a previously described mutation in HSAN2B and shows reduced mRNA level of the mutated gene. This mRNA decay has been speculated previously but not been shown. Identification of* FAM134B *mutation will be helpful for identifying carriers in the extended members of these families for genetic counseling.

## Figures and Tables

**Table 1:** Summary of the clinical and neurophysiological findings.

**Individual as indicated in Figures 1(a) and 1(b)**	**Sex / Age**	**Silent Clinical Features**	**Motor Study**	**Sensory Study**	**Comment**
**Family 1: IV-1**	F/10	Delayed walking, spastic gait, foot ulcers	Median nerve CMAP/AMP = 9.2 (N ≥ 8 mV) Median nerve CMAP/CV = 46 (N ≥ 45 m/s) Peroneal nerve CMAP/AMP = 3.3 (N ≥ 3.0 mV) Peroneal nerve CMAP/CV = 46 (N ≥ 42 m/s)	Median nerve SNAP/AMP = 10.2 (N ≥ 9.5 mV) Median nerve SNAP/CV = 55 (N ≥ 53 m/s) Sural nerve SNAP/AMP = 8.5 (N ≥ 6 mV) Sural nerve SNAP/CV = 45 (N ≥ 48 m/s)	Low normal Normal autonomic studies (beat to beat variation and sympathetic skin response)

**Family 1: IV-1** **(Repeated study)**	F/28	Multiple foot and leg ulcers with mutilating acropathy	Median nerve CMAP/AMP = 6.1 (N ≥ 8 mV) Median nerve CMAP/CV = 42 (N ≥ 45 m/s) Peroneal nerve CMAP/AMP = 2.5 (N ≥ 3.0 mV) Peroneal nerve CMAP/CV = 35 (N ≥ 42 m/s)	Median nerve SNAP/AMP = 3.0 (N ≥ 9.5 mV) Median nerve SNAP/CV = 45 (N ≥ 53 m/s) Sural nerve SNAP/AMP = 0.5 (N ≥ 6 mV) Sural nerve SNAP/CV = 37 (N ≥ 48 m/s)	Repeated study confirmed severe sensory axonal polyneuropathy in length dependent fashion

**Family 1: IV-2**	M/23	Delayed walking, spastic gait, repeated falls, foot and hand ulcers	Ulnar nerve CMAP/AMP = 6 (N ≥ 7 mV) Ulnar nerve CMAP/CV = 42 (N ≥ 45 m/s) Peroneal nerve CMAP/AMP = 3.2 (N ≥ 3.0 mV) Peroneal nerve CMAP/CV = 46 (N ≥ 42 m/s)	Ulnar nerve SNAP/AMP = 2.1 (N ≥ 9.5 mV) Ulnar nerve SNAP/CV = 46 (N ≥ 55 m/s) Sural nerve SNAP/AMP = absent (N ≥ 6 mV) Sural nerve SNAP/CV = absent (N ≥ 48 m/s)	Axonal sensory more than motor polyneuropathy

**Family 2: IV-1**	M/10	Repeated falls, spastic gait and multiple foot ulcers started at age 5	Median nerve CMAP/AMP = 10.2 (N ≥ 8 mV) Median nerve CMAP/CV = 55 (N ≥ 45 m/s) Peroneal nerve CMAP/AMP = 3.2 (N ≥ 3.0 mV) Peroneal nerve CMAP/CV = 47 (N ≥ 42 m/s)	Median nerve SNAP/AMP = 9.4 (N ≥ 9.5 mV) Median nerve SNAP/CV = 55 (N ≥ 53 m/s) Sural nerve SNAP/AMP = 7.5 (N ≥ 6 mV) Sural nerve SNAP/CV = 50 (N ≥ 48 m/s)	Low normal Normal autonomic studies (beat to beat variation and sympathetic skin response)

CMAP = compound muscle action potential; AMP = amplitude; CV = conduction velocity; SNAP = sensory nerve action potential; mV = millivolt; *μ*V = microvolt; m/s = meter per second.

## Data Availability

All data are contained within the article.

## Abbreviations

HSANs: Hereditary sensory and autonomic neuropathies
HSAN2B: Hereditary sensory and autonomic neuropathy type IIB
ROH: Region of homozygosity
WES: Whole-exome sequencing.
